# Advancing TB research using digitized programmatic data

**DOI:** 10.5588/ijtld.21.0325

**Published:** 2021-11-01

**Authors:** J. Taaffe, J. Croda, H. Moultrie, D. S. Silva, A. Rosenthal, M. Farhat

**Affiliations:** 1 Office of Cyber Infrastructure and Computational Biology, Department of Health and Human Services, National Institute of Allergy and Infectious Diseases, National Institutes of Health, Bethesda, MD, USA; 2 Federal University of Mato Grosso do Sul, Campo Grande, MS, Brazil; 3 Department of Epidemiology of Microbial Diseases, Yale University School of Public Health, New Haven, NJ, USA; 4 Oswaldo Cruz Foundation, Campo Grande, MS, Brazil; 5 National Institute for Communicable Diseases, Division of the National Health Laboratory Service, Johannesburg, South Africa; 6 Sydney Health Ethics, University of Sydney School of Public Health, Sydney, NSW, Australia; 7 Department of Biomedical Informatics, Harvard Medical School, Boston, MA; 8 Division of Pulmonary and Critical Care, Massachusetts General Hospital, Boston, MA, USA

**Keywords:** digital health, data sharing, TB programs

## Abstract

The use of real-world data from national TB care programs has great potential to answer key research questions in TB control and is now opportune due to increasing digital data collection and storage. We summarize an expert stakeholder workshop conducted on this topic in October 2019, with perspectives from academics, national TB program officers, and data managers. We discuss challenges and opportunities in the use of TB programmatic data for research and describe digital data availability in two large, high TB burden countries, Brazil and South Africa. From this, we posit that with a standardized data collection set, improved data management, and greater collaboration, more TB programmatic data can be used for research with measurable public health impact.

## Abstract (FR)

L’utilisation de données en vie réelle, issues des programmes nationaux de lutte contre la TB, est grandement susceptible de répondre aux questions de recherche clés relatives au contrôle de la TB. L’utilisation de ces données s’impose aujourd’hui au vu du nombre croissant de données numériques recueillies et stockées. Nous synthétisons ici un atelier de travail ayant réuni des parties prenantes expertes en la matière en octobre 2019, avec le point de vue d’universitaires, d’administrateurs de programmes nationaux de lutte contre la TB et de gestionnaires de données. Nous abordons les défis et opportunités liés à l’utilisation des données des programmes de lutte contre la TB à des fins de recherche et décrivons la disponibilité des données numériques dans deux grands pays à forte incidence de TB (Brésil et Afrique du Sud). Nous affirmons qu’en standardisant le recueil des données, en améliorant leur traitement et en renforçant la collaboration, davantage de données issues des programmes de lutte contre la TB pourront être utilisées à des fins de recherche avec un impact mesurable sur la santé publique.

The WHO Global TB Program has been advocating and supporting wider and more detailed data collection on TB care for decades.^1^,^2^ Since 2009, the availability of robust TB burden data from both routine TB programmatic data collection and systematic surveys of TB disease burden and antibiotic resistance prevalence has significantly increased.^1^–^4^ More recently, pathogen whole-genome sequencing (WGS) is being generated for the detection of resistance and surveillance, and for tracking disease transmission in low TB prevalence settings.^5^

Programmatic data originate from the real-world care of large populations of TB cases. Real-world data have the potential to provide generalizable answers to important questions that span implementation, epidemiology, and clinical research for improved disease control—as long as it is sufficiently detailed to allow control for important confounders.^3^ It can also support basic research on human susceptibility to infection, treatment cure, and pathogen biology. The reuse of this expanding digital TB data is thus valued by global health funders, implementers, and scientists, with echoing calls for improved data quality, data sharing, and collaboration between stakeholders (Figure).^1^

**Figure:**
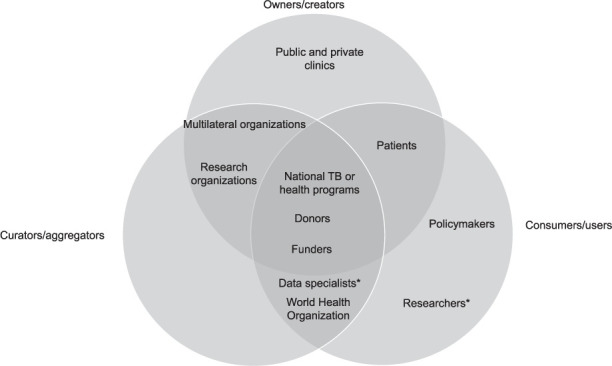
Schematic of TB programmatic data stakeholders. ^*^Including bioinformaticians.

At the 2019 Union Conference in Hyderabad, India, we convened an expert panel to discuss the potential and barriers facing the use of programmatic data for TB research. The meeting’s participants included major stakeholders on the topic, representing the diverse community of groups that own, curate, or consume TB data (Figure, Acknowledgements). We share here a summary of the major points raised in the meeting, a set of core data variables relating to two research use cases, and an agreed way forward to enable usage of TB programmatic data for research.

## KEY OPPORTUNITIES FOR RESEARCH WITH TB PROGRAMMATIC DATA

As the opportunities for research with programmatic data is wide, we focus on two specific use cases:

### TB treatment outcomes

An important use case is the identification of predictors of TB treatment outcome. TB treatment is long and complex, with varied outcome across programs and populations due to differing biological, clinical, and socio-behavioral factors.^6^,^7^ An improved understanding of treatment outcome predictors across real-world scenarios can help TB programs avoid the current one-size-fits-all approach to TB treatment. It can also facilitate optimal use of expensive and limited laboratory resources such as mycobacterial culture that can confirm bacteriological clearance and monitor treatment response.

### Molecular resistance diagnosis

A second use case is research to improve antibiotic resistance detection using DNA mutation detection or sequencing technology, a faster and more cost-effective solution than mycobacterial culture. There is a limited spectrum of antibiotics reliably testable with this approach and an increasing number of mutations of unknown significance for predicting treatment response. Further research on how specific resistance mutations relate to treatment outcome is needed, including those that are relatively rare and variably distributed geographically but impactful, such as the I491F *rpo*B mutation.^8^

## PROGRAMMATIC DATA COLLECTED BY THE WHO, BRAZIL, AND SOUTH AFRICA

Individual patient-level clinical data are routinely generated during TB care, increasingly digitized, and variably linked to laboratory data such as molecular resistance detection. National TB programs (NTPs) use this information for surveillance and reporting purposes. The majority of TB data currently remain decentralized, with a handful of key data elements and indicators shared through the WHO’s Global TB Database.^9^ These reports specifically include country-level burden estimates for drug-susceptible and drug-resistant TB case notifications by age, sex, localization (pulmonary or extrapulmonary), prior treatment history, TB-HIV coinfection rates, as well as treatment outcomes.

Brazil and South Africa are among the top 30 highest TB burden countries globally and are both emerging economies with consistent domestic investment in TB surveillance and care.^2^ Representatives of Brazil and South Africa’s TB programs provided an overview of their data collection process and infrastructure at the workshop. The Brazilian NTP collects data electronically at the individual patient level, including socio-demographics, prior TB history, TB localization, pregnancy status, comorbidities, treatment dates and regimen, outcomes, contact information, laboratory results, and chest X-ray interpretation. These data are stored across several databases, but an anticipated national decree will require a unique health identifier to facilitate data linkage. De-identified Brazilian health data are accessible to the public from the Notifiable Health Conditions Information System (*Sistema de Informação de Agravos de Notificação*, SINAN).^10^ In South Africa, TB clinical/treatment data and laboratory data are stored electronically in separate databases. Clinical data include basic demographic data, pregnancy status, TB treatment dates and regimen, and HIV and antiretroviral therapy status. Laboratory data include routine GeneXpert testing and line-probe results. Similar to Brazil, the lack of a South African unique health identifier is acknowledged by curators as a major barrier in data linkage, with estimated under-match linkages of approximately 10–15%.^11^ Aggregated laboratory data, including the frequency of drug-susceptible and -resistant cases are publicly available through a dashboard,^12^ which can stratify the data by age, sex, and province. Detailed data can also be downloaded through a restricted access dashboard with permission from the National Institute for Communicable Diseases, Johannesburg, South Africa.

## CHALLENGES IN THE RESEARCH USE OF TB PROGRAMMATIC DATA

Using programmatic data for research requires that necessary variables are available and reliably coded. We pre-specified a set of important data variables relevant to use cases 1 and 2 and solicited input on their availability from the WHO and programmatic representatives from Brazil and South Africa (Table). The WHO receives and aggregates data on 12 of the 30 pre-specified variables.^9^ Brazil and South Africa’s NTP collect data on respectively 16 of 30 and 15 of 30 pre-specified variables, and partial data on an additional six and five variables. Missing variables reflect assessments of comorbid disease, adverse events related to drug treatment, and treatment adherence. Digitized data on routine laboratory tests such as GeneXpert are available, along with geographic and temporal tags, but linkage to the individual patient record is challenged by the use of different database identifiers. Mycobacterial culture and culture-based drug susceptibility (DST) data are largely restricted to patients with recognized or suspected rifampicin resistance in Brazil and South Africa. While increasingly generated for research in Brazil and South Africa, TB WGS is not yet routinely generated to guide patient management or linked to patient level characteristics in either country.

**Table:**
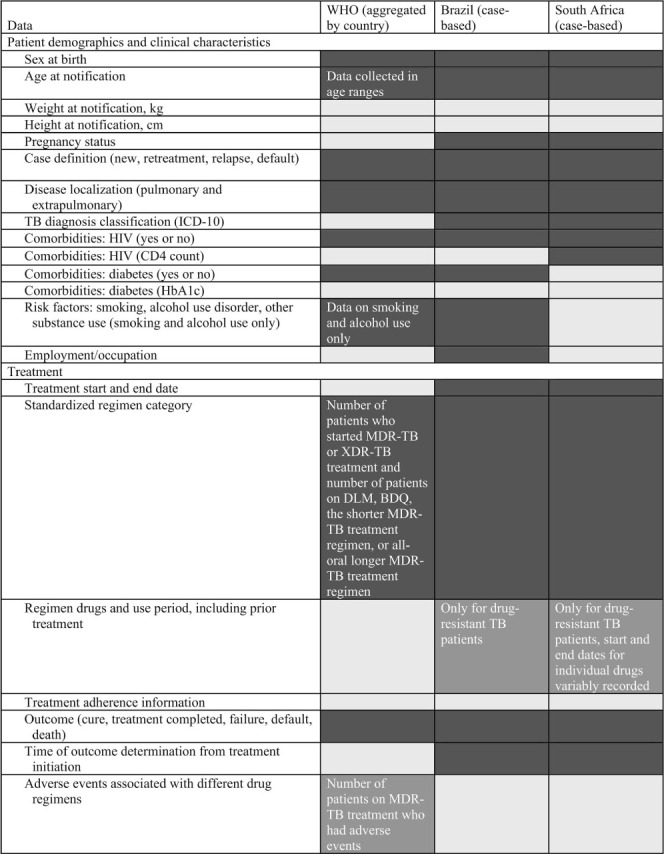
Key data variables collected by WHO and Brazilian and South African NTPs

**Table:**
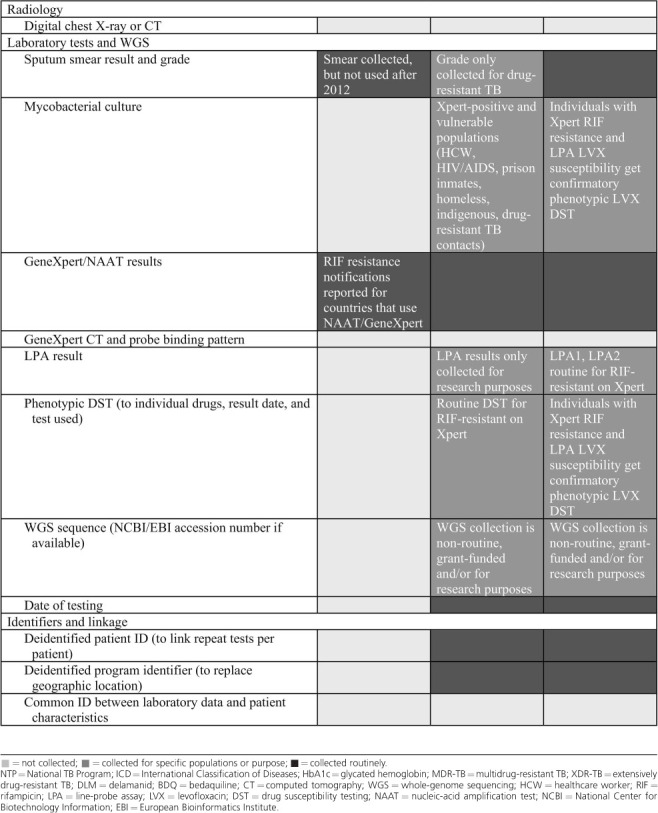
(*continued*)

Data quality, standardization, and accessibility are key enablers of research. Some NTPs continue to collect data in paper-based systems that are aggregated periodically for central-level reporting. Errors can be difficult to detect or correct due to challenges in accessing individual case records. The use of aggregate paper reporting is also a major barrier to accessibility and reuse of data for research. Electronic case-based systems are favored, but also present their own technological challenges. There may not be protocols or staff in place for ensuring data quality, e.g., independent data entry checks or rejection of entries that fail to meet strict field definitions. Inadequate training, turnover of program staff, and misinterpretation of definitions lead to inaccuracies or incomplete case records. Electronic data collection and storage systems, like the open-source and WHO-supported District Health Information System (DHIS2), are increasingly adopted to address these gaps. However, limited technical infrastructure in many low- and middle-income countries, particularly in rural areas, are still barriers to nationwide roll out.

## SHARING PROGRAMMATIC DATA ETHICALLY

A major concern is ensuring the privacy, confidentiality, and security of patient data. This will require the community of TB stakeholders to consider what sharing of programmatic data will mean in application, especially if global data repositories are generated. Particular consideration should be given to protecting any administrative data that could identify vulnerable populations. The regulatory landscape around data sharing is also actively evolving. In South Africa, organizations must fully comply with sections of the Protection of Personal Information Act (PoPIA) as of July 2021. The implications for international sharing of anonymized health data will become clearer once the health and/or research code of conduct has been promulgated there. Sharing data ethically also requires trust. Patients need to trust NTP staff and researchers with their private information. Research shows that given the complexity of big programmatic data, especially from advanced laboratory diagnostics or next-generation sequencing, treating clinicians will have difficulty understanding how results come about.^13^ Thus, trust must be built between patients, clinical and laboratory providers, and researchers.

## MOVING AHEAD

In an era of increasing digitization of healthcare data, the use of TB programmatic data for research is now feasible but must be prioritized by stakeholders to ensure quality and success. Here, we provide a standardized dataset with variables relevant for TB care and surveillance research to guide national TB data collection. We find that a substantial number of the variables we designate as important is routinely collected in the two programs we highlight. Key gaps include information on comorbid disease, TB treatment adherence, adverse effects, and select diagnostic data. We identify data management as a challenge to quality and accessibility. Training and digitalization tool kits should include variables lists, definitions, data standards, infrastructure such as the DHIS2 and best practices for data collection. Implementation of a unique identifier for data linkage is also critical.

Moving forward requires close collaboration between all stakeholders. Data specialists and IT professionals can help address challenges with data quality and management, working directly with those collecting data locally. Researchers and policymakers must engage with data owners, including TB patient groups, to maximize the usage of data and knowledge gained. Policymakers can also advocate for responsible data linkage and sharing policies. Funders can incentivize efforts in digitization and standardization; along with multilateral organizations, they may even become partners in developing and managing TB repositories. Ethically, task forces with broad stakeholder representation should guide how data are shared to enable research, keeping in mind privacy concerns vs. research trade-offs.

There is good precedent for the successful and wide sharing of clinical data for research. The WHO is employing a new approach to coordinate individual participant data (IPD) through data curators, aimed at improving data quality and access through a digitization drive. The UK Biobank shows how standard data use agreements that set minimum data management and usage requirements can be developed to build trust and facilitate data exchange between data owners and utilizers.^14^ Cloud-based solutions can also facilitate greater access to and usage of data,^15^ and platforms and tools such as the National Institute of Allergy and Infectious Diseases TB Portals, GenTB (Translational Genomics platform for TB), and ReSeqTB (Relational Sequencing TB Data Platform) share data and tools that support translational clinical research.^16^–^18^ Furthermore, digitalization should link already existing contributions, such as the Global TB Network (GTN) (active in both Brazil and South Africa), who go on to collect missing data on active TB drug safety monitoring (aDSM),^19^,^20^ and to investigate possible interaction between COVID-19 and TB.^21^

With greater will and support, much more is possible. The incredible speed at which the scientific and public health communities have moved to openly share and analyze COVID-19 data is a testament to what can be achieved when the need is urgent.^22^ For TB patients and physicians wanting shorter, more effective treatments, for public health practitioners trying to control drug-resistant TB, and for researchers seeking real-world data to work on these problems, the need is urgent.
